# Investigating the knowledge, perception, and practice of healthcare practitioners toward rational use of compounded medications and its contribution to antimicrobial resistance: a cross-sectional study

**DOI:** 10.1186/s12913-022-07649-4

**Published:** 2022-02-23

**Authors:** Desta Assefa, Getahun Paulos, Dereje Kebebe, Sintayehu Alemu, Wondu Reta, Temesgen Mulugeta, Fanta Gashe

**Affiliations:** School of Pharmacy, Jimma University, Oromia, Ethiopia

**Keywords:** Antimicrobial resistance, Compounding, Healthcare practitioners, Rational use

## Abstract

**Background:**

Pharmaceutical compounding ensures access of individuals with specific requirements to individualized therapy. However, there is an inconsistency of compounded medication quality. Therefore, advancing the rational use of compounded medication is essential for patient safety and medication effectiveness.

**Objective:**

The presented study was aimed to investigate the healthcare practitioners’ knowledge, perception, and practice of extemporaneous compounding and its contribution to the prevalence of antimicrobial resistance.

**Method:**

A descriptive cross-sectional survey using a structured questionnaire was conducted. The study participants were 300 healthcare practitioners working in Jimma University Medical Center, hospital pharmacies, and community pharmacies in Jimma and Mettu Town, Southwest Ethiopia.

**Results:**

Most respondents were pharmacists (62.7%) and first-degree holders (48.3%). The majority of them had experience in administering (57.7%), preparing (38%), prescribing (21%), and repackaging and labeling (14%) compounded medications. Commonly they request compounded medications when prepackaged products (77.7%) and needed dosage regimens (72.3%) were not available in the market. However, most of them believed that compounded medications might lack quality (49%) and had poor patient compliance (40.7%). Moreover, they fear that inappropriate preparation processes (75%) and under-dose administration (59%) of compounded medication might contribute to the development and prevalence of antimicrobial resistance.

**Conclusion:**

Most healthcare practitioners practice rational use of compounded medications and strongly agree that inappropriate compounding of antimicrobials contributes to antimicrobial resistance development.

## Introduction

Ensuring medication availability in healthcare facilities (HCFs) is crucial to providing good quality healthcare and reducing avoidable patient readmissions. In addition to manufacturing in the licensed pharmaceutical industries, extemporaneous compounding is the essential source of medication availability, especially for personalized healthcare [1–4].

Since the mid-1980s, the number of patients seeking personalized healthcare has been increasing. It is due to one size medicine (strength and dosage form) does not fit all. Therefore prescribing, dispensing, and administering extemporaneously compounded medication is a good solution. Extemporaneous compounding is the art and science of combining, mixing, repackaging, and labeling a drug product. Historically, it has been a crucial part of the pharmaceutical process. It has long been a service provided by licensed pharmacists since the origin of pharmacy. Today, with the technological advancements in the pharmaceutical field and increased need, the compounding pharmacy industry is experiencing a resurgence and becoming more relevant than ever [3, 5–11].

The medicine compounders compound according to a licensed practitioner’s prescription, medication order, or initiative based on the practitioner-patient-pharmacist relationship in the course of professional practice [1, 12]. It includes removal of allergenic ingredients, dosage form change, especially for easy swallow, altering strength, routes of the delivery, unpleasant flavor of available drug product, and allowing access to discontinued medications [5–7, 9, 12–14]. Therefore, compounding allows specific patients such as pediatric and geriatric patients, patients requiring dermatologic disease treatment and pain management, and others with more options [1, 8].

The compounded medications should be developed to meet unique medical needs and ensure patient safety. Therefore, compounding prescriptions should be prescribed by a licensed practitioner, compounded and dispensed by the licensed pharmacist, administered and used correctly. Compounding pharmacies must also comply with good compounding practice regulations for compounding within hospitals, community pharmacies, home infusion pharmacies, and, more recently, outsourced compounding facilities [1, 8, 9].

The regulatory scrutiny of pharmaceutical compounding practices is significantly less rigorous than that required for FDA-approved medications (commercially available medications). It is exempted from regular inspections, quality control testing, and rejection of material not meeting specifications. Furthermore, they are not clinically assessed for safety or efficacy before marketing. Moreover, there is no standard labeling or prescribing information with instructions for the safe use of compounded products. They are the potential causes for poor quality medication preparation (contamination, medication with too little or too much active ingredient, or failure to meet specifications). Finally, they may pose potentially fatal health risks for the users [5, 6, 12, 15–19]. As evidence, FDA reported the prevalence and potential risks of poor quality compounded medications many times. These defects lead to serious patient illnesses, adverse events, and deaths linked to poor quality compounded drugs [16, 20, 21].

Poor quality of compounded medication causes administration of a low dose of active ingredients. Administration of lower-dose antimicrobials can cause microbes, including bacteria, fungi, parasites, and viruses, to adapt and become less susceptible to medical treatment. Therefore, it can play a role in the emergence and spread of antimicrobial resistance (AMR), which causes a profound threat to human health. Moreover, irrational prescribing, packaging and labeling, administering, and using compounded medicine can contribute to AMR prevalence [1, 22–24]. Thus, the rational use of compounded antimicrobials can be the critical action to prevent the spread of antimicrobial-resistant infections. It mostly depends on healthcare practitioners' (HCPs') knowledge, perceptions, and practices. The presented study was aimed to investigate their knowledge, perception, and practice of extemporaneous compounding and its contribution to the prevalence of AMR.

## Methods

### Study design and settings

A descriptive cross-sectional study was conducted from March 15 to May 25, 2021, among the HCPs working in selected HCFs. The HCFs were Jimma University Medical Center, hospital pharmacies, and community pharmacies located in Jimma Town and Mettu Town. These towns are located at 346 km (Jimma Town) and 600 km (Mettu Town) from Addis Ababa, the Capital City of Ethiopia in the Southwest. The study settings have been giving healthcare services to a large population. They were selected through convenience sampling.

Jimma University Medical Center is the only teaching and referral hospital in the southwestern part of Ethiopia. With 800-bed capacity and hospital pharmacies, it provides healthcare services for inpatient and outpatient attendants coming to the hospital from the catchment population. Moreover, community pharmacies in study settings are giving pharmaceutical care. It includes compounding pharmaceutical products and dispensing.

### Study participants and sampling method

The study participants were HCPs such as physicians, pharmacists, nurses, medical laboratory technologists, and midwives. They were chosen since the study focused on one of the most important elements of healthcare. So, they should be aware of the need for compounded medications and their safety. Additionally, they should be encouraged to report potential risks linked to poor quality compounded drugs and effectively protect the patients.

The voluntary study participants were selected and sampled using the convenience sampling technique. Accordingly, 243 HCPs from Jimma Town and 57 (19%) pharmacists from Mettu Town were involved in the study. Due to pharmacy professionals being formally licensed and experienced professionals for compounding both sterile and non-sterile medications [1, 25], most of the participants (188 (62.7%) were pharmacists.

### Data collection

Data was collected using a self-administered structured questionnaire. It was constructed using the English language after an intensive review of published relevant literature [24, 26, 27]. The developed questionnaire had three sections containing closed-ended questions. The first section asked about the respondents' demographic characteristics. The second section described their experience of practicing rational use of extemporaneously compounded antimicrobial medications, reasons for performing antimicrobial compounding, and not practicing their rational use. The third section asked about the perception and knowledge of the respondents on the contributions of extemporaneously compounded antimicrobials to the development of antimicrobial resistance.

A pilot study was conducted using the first draft of the survey instrument. Randomly selected four respondents from each HCF have filled the questionnaire and commented on the clarity of each question. Their comments and responses were evaluated and used to guide revisions. Finally, minor editions were performed to avoid the ambiguity of the questionnaire. Data obtained from the pilot study was not included in the final analysis.

The principal and co-investigators provided training for data collectors (pharmacists and nurses). Before data collection, potential study participants were verbally provided all pertinent information about the study. After allowing them ample opportunity to ask for uncertainty, data collectors provided the data collection sheet. Following sufficient time for reading, the study participants were asked for their voluntarism and requested to agree to verbal informed consent before filling the questionnaire. The data collector supplies the questionnaire for the voluntary participant and collects it after fill. The collected data were checked for completeness and de-identified to ensure confidentiality.

### Data analysis

After collecting the filled questionnaire, data were entered and descriptively analyzed using Statistical Package for the Social Sciences (SPSS), version 21.0. Descriptive analysis, such as frequency distribution and percentage, was used to analyze categorical data.

**Inclusion criteria:** Voluntary HCPs on duty during the study period were contacted and included in the study.

**Exclusion criteria:** Involuntary HCPs were excluded from the study.

### Operational definition

Extemporaneous compounding: Small scale preparation, altering, mixing, repackaging, and labeling of medication with prescriber’s instructions [1].

Rational use of compounded medication: Compounded medication accomplishing key elements of rational drug use such as effective, acceptable quality, acceptable safety, accurate prescribing (drug, dose, interval, and duration), appropriate and timely administration, affordable and dispensed correctly for the right patient [28].

## Results

The present study was conducted using 300 study participants who were voluntarily consented to participate in the study. They were working at different units at Jimma University Medical Center, such as hospital pharmacies and wards (148), Jimma Town community pharmacies (95), and Mettu Town community pharmacies (57). The respondents had worked as healthcare professionals for a mean of 5.78 ± 4.6 years (range 1–32 years) (Table 1).

**Table 1:** Demographic characteristics of healthcare practitioners in the healthcare facilities

**Characteristics**		**N**o. **= 300, 100**%	**Characteristics**		No. = 300, 100%	
Sex	Male	176 (58.7)	Respondent profession	Physician	28 (9.3)	
	Female	124 (41.3)		Medical laboratory	24 (8)	
Age (years)	20–30	175 (58.3)		Nursing	54 (18)	
	30–35	66 (22)		Pharmacy	188 (62.7)	
	35–40	23 (7.7)		Midwives	6 (2)	
	> 40	36 (12)				
Educational level	Diploma	142 (47.3)	Work experience (years)	< 5	151 (50.3)	
	First degree	145 (48.3)		5–10	92 (30.7)	
	Specialty (Master degree)	13 (4.4)		> 10	57 (19)	
			Took training on compounding medications			73 (24.3%)

The present study indicated that all the HCPs mentioned different reasons for requesting and performing compounding medication and their route of administration (Table 2).

**Table 2:** Reasons for performing extemporaneous compounding of medications and route of administration

Reasons for performing extemporaneous compounding of medicine	No. = 300, 100%
Unavailability of prepackaged products in the market	233 (77.7)
Unavailability of needed dosage regimen (dose, form) (individualized therapy)	217 (72.3)
The product was discontinued by the manufacturer	81 (27)
To combine two or more active ingredients into one product	128 (42.7)
To improve adherence to medications (flavor, taste)	68 (22.7)
Allergy to the excipient(s) in the commercially available product	87 (29)
Compounded medications are less expensive	90 (30)
Ministry of Health encourages compounding	65 (21.7)
Improving the stability of the desired product	61 (20.3)
**Route of administration for compounded medication**	
Oral	214 (71.3)
Ophthalmic	148 (49.3)
Topical (Skin)	121 (40.3)
Parenteral	89 (29.7)
Otic	82 (27.3)
Rectal	44 (14.7)
Nasal	32 (10.7)
Inhalation	24 (8)
Rectal	44 (14.7)
Others	5 (1.7)

**Fig. 1:**
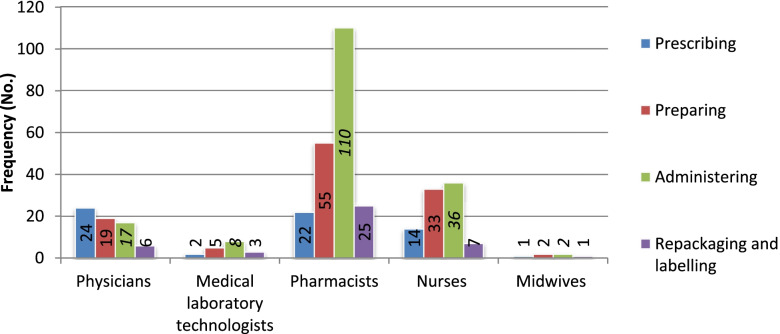
Actual practicing of the rational use of extemporaneously compounded medications

Multiple response analysis indicated that 262 (87.33%) of the study participants had participated in at least one process of rational use of extemporaneously compounded medications (Fig. 1). They include administering 173 (44.1%%), preparing 114 (29.1%), prescribing 63 (16.1%), and repackaging and labeling of compounded medications 42 (10.7%), respectively. 91% of trained respondents on extemporaneous compounding of medication had practiced compounding services, especially administering (44, *p* = 0.60 and preparing (30) (*p* = 0.53). Most first-degree and specialty (master degree) holders had practiced more on prescribing (47), preparation (75), and repackaging and labeling (27) of compounded medications. However, most diploma holders (91) had practiced administration of compounded medications.

The study respondents practiced rational use of extemporaneously compounded antimicrobials at different frequencies. Most of them practiced like monthly (occasionally) 108 (36%), almost every day 93 (31%), not at all 77 (25.7%) and always 22 (7.3%), respectively. Most of the time, the respondents didn’t prescribe, dispense or prepare antimicrobial medicine extemporaneously due to different challenges (Table 3).

**Table 3:** Challenges to practice rational use of extemporaneously compounded antimicrobial medicine

Challenges		No. = 300, 100%
Lack of trust in the quality of the compounded formulations		147 (49)
Belief that compounded formulations have poor patient compliance		122 (40.7)
Compounded prescriptions are inconvenient economically		49 (16.3)
Belief that the ministry of health does not allow compounded formulations		33 (11)
not adequate	skilled personnel	49 (16.3)
	manufacturing environment	80 (26.7)
	no ingredients	50 (16.7)
Fear of inappropriate use (overuse or underuse)		67 (22.3)
Fear of AMR development		73 (24.3)
other (specify)		3 (1)

The present study showed the perception of HCPs on the development of AMR due to antimicrobial compounding. Moreover, most of them have knowledge concerning the compounding factors contributing to AMR development (Table 3).

## Discussion

Pharmaceutical compounding has long been an essential component of health care. So, rational use of compounded medications is a fundamental activity within HCFs (9). The main findings of the present investigation showed that most of the HCPs participated in the preparation and administration of the compounded medications (Fig. 1). Pharmacy professionals were the most common practitioners. It might be due to pharmacists being formally trained in the art and science of compounding medications in their undergraduate courses and licensed to compound medicine relative to other medical communities [1, 23]. Moreover, a limited number of respondents took training on the compounding of medications.

An earlier study has linked the prevalence of compounding services to requests for individualized therapy [29, 30]. Similarly, the present study showed that the most common reasons for requesting and providing compounding services are the unavailability of FDA-approved drugs in the prepackaged form in the market and the needed dosage regimen for individualized therapy (Table 2). Moreover, the availability of requested dosage regimens for individualized therapy contributes to users' adherence and therapeutic efficacy.

Various studies indicated the high demand for pharmaceutical compounding services at different levels of HCFs. However, the service is not prevalent due to several challenges [10, 31]. Accordingly, the present study reported HCPs' lack of trust in the quality of the compounded formulations and fear of poor patient compliance as common challenges (Table 3). This finding agreed with a study conducted by Abdel Naser Zaid et al. (2010) [31]. It can be due to the quality of compounded products is not regulated by the Ethiopian FDA. Moreover, patients who suffer from the side effects of the compounded medication may bring negligence and malpractice claims against HCPs [16]. As a result, HCPs should be careful during providing rational use of compounded products.

Previous studies reported a lack of prescriptions for requesting compounding services as one of the key reasons for not providing compounding services (27,32). However, the present study did not. It might be due to the prescribers' lack of confidence in the quality and effectiveness of the compounded medication.

Extemporaneously compounded medications are not undergoing regular inspections, quality control testing, and rejection of material not meeting specifications. However, their quality should be regulated to ensure safety [12]. The present study showed that most respondents strongly believed that substandard extemporaneous preparation of antimicrobials contributes to AMR development. They mentioned inappropriate preparation processes, under-dose administration, and contamination during compounding as the factors causing AMR development (Table 4). The responses agreed to the fact that a low dose of active ingredients in poor quality of compounded medications causes microbes to adapt and become less susceptible [1, 19–21].

**Table 4:** Perception and knowledge of healthcare practitioners on the contributions of antimicrobial compounding to antimicrobial resistance

Statements for perception evaluation		N^o^. = 300, 100%					
		strongly agree	Agree	Neutral	Disagree	Strongly disagree	Don’t know
More cautious use of antimicrobials in extemporaneous formulations would decrease AMR		93 (31)	95 (31.7)	45 (15)	6 (2)	21 (7)	40 (13.3)
For extemporaneous compounding, using broad-spectrum antimicrobials can be used in place of narrow-spectrum antimicrobials to reduce resistance		37 (12.3)	82 (27.3)	101 (33.7)	46 (15.3)	12 (4)	22 (7.3)
Inappropriate or substandard extemporaneous compounding of antimicrobials contributes to AMR development		112 (37.3)	46 (15.3)	49 (16.3)	11 (3.7)	20 (6.7)	62 (20.7)
**Statements for knowledge evaluation**		**N**o** = 300, 100%**					
Factors contribution for AMR development	Under dose administration	177 (59)					
	Contamination	86 (28.7)					
	Inappropriate preparation process due to lack of standard operating procedures	225 (75)					
	Ineffectiveness nature of compounded preparation	47 (15.7)					
	Incompatibility between drug and excipient(s)	46 (15.3)					
	Using after beyond use date	44 (14.7)					
	Other (specify)	2 (0.7)					

## Conclusions

The present study indicated that most healthcare practitioners provide healthcare services using compounded medications. They apply rational use principles mainly for administering and preparing compounded medications. Unavailability of prepackaged products in the market and unavailability of needed dosage regimens were the most common reasons mentioned for practicing rational use of compounded medication. However, lack of trust in the quality of the compounded medication and poor patient compliance was the most listed challenges. The majority of them revealed that compounding caused inappropriate preparation processes, and under-dose medication administration can lead to the development and prevalence of antimicrobial resistance.

## Recommendation

Due to individualized therapy is being in high demand, compounding services are becoming critical. Thus, healthcare practitioners should take training on extemporaneous compounding. Moreover, compounding services should be incorporated into professional development and continuing education programs, and the quality of compounded medication should be regulated.

## Limitation of the study

The present study did not statistically analyze the degree of correlation between challenges and reasons of compounding to compounding practices. It can be a basic study point for future researchers.

## Data Availability

Data and supplementary materials are readily available from the corresponding author on reasonable request.

## Abbreviations

AMR: Antimicrobial resistance
HCP: Healthcare practitioner
HCF: Healthcare facility
